# Validation of the Palliative Prognostic Index, Performance Status–Based Palliative Prognostic Index and Chinese Prognostic Scale in a home palliative care setting for patients with advanced cancer in China

**DOI:** 10.1186/s12904-020-00676-0

**Published:** 2020-10-31

**Authors:** Jun Zhou, Sitao Xu, Ziye Cao, Jing Tang, Xiang Fang, Ling Qin, Fangping Zhou, Yuzhen He, Xueren Zhong, Mingcai Hu, Yan Wang, Fengjuan Lu, Yongzheng Bao, Xiangheng Dai, Qiang Wu

**Affiliations:** 1 Department of Spine Surgery, Yuebei People’s Hospital Affiliated to Shantou University Medical College, Shaoguan, Guangdong China; 2 Department of Nursing, Yuebei People’s Hospital Affiliated to Shantou University Medical College, Shaoguan, Guangdong China; 3 Hospice center of Yuebei People’s Hospital Affiliated to Shantou University Medical College, Shaoguan, Guangdong China; 4 Emergency rescue command center of Shaoguan city, Shaoguan, Guangdong China; 5 Hospice center of Fourth Affiliated Hospital of Guangxi Medical University, Liuzhou, Guangxi China; 6 Department of Spinal Surgery, Nanfang Hospital, Southern Medical University, Guangzhou, Guangdong China

**Keywords:** Validation, Palliative prognostic index, Performance status–based palliative prognostic index, Chinese prognosis scale, Advanced Cancer, Home-based palliative care setting

## Abstract

**Background:**

The predictive value of the prognostic tool for patients with advanced cancer is uncertain in mainland China, especially in the home-based palliative care (HPC) setting. This study aimed to compare the accuracy of the Palliative Prognostic Index (PPI), the Performance Status–Based Palliative Prognostic Index (PS-PPI), and the Chinese Prognosis Scale (ChPS) for patients with advanced cancer in the HPC setting in mainland China.

**Methods:**

Patients with advanced cancer admitted to the hospice center of Yuebei People’s Hospital between January 2014 and December 2018 were retrospectively calculated the scores according to the three prognostic tools. The Kaplan-Meier method was used to compare survival times among different risk groups. Receiver operating characteristic curve analysis was used to assess the predictive value. The accuracy of 21-, 42- and 90-day survival was compared among the three prognostic tools.

**Results:**

A total of 1863 patients were included. Survival time among the risk groups of all prognostic tools was significantly different from each other except for the PPI. The AUROC of the ChPS was significantly higher than that of the PPI and PS-PPI for 7-, 14, 21-, 42-, 90-, 120-, 150- and 180-day survival (*P* < 0.05). The AUROC of the PPI and PS-PPI were not significantly different from each other (*P* > 0.05).

**Conclusions:**

The ChPS is more suitable than the PPI and PS-PPI for advanced cancer patients in the HPC setting. More researches are needed to verify the predictive value of the ChPS, PPI, and PS-PPI in the HPC setting in the future.

## Background

It is one of the basic parts of palliative care to predict the survival of patients with advanced cancer accurately [1, 2]. In the final stages of a cancer patient’s life, predictions of survival will help the patient and family decide whether to continue treatment and help achieve patients’ last wishes [1, 3–5]. Clinical prediction of survival tends to overestimate the actual survival time of advanced cancer patients because clinical prediction of survival is based on the experience of physicians [6–8]. A previous study revealed that predicting with the application of prognostic tools can provide the patient and family with more accurate prognostic information [9].

Prognostic tool plays an important role in palliative care, but the study about prognostic tools for advanced cancer patients is still in its infancy in mainland China [10, 11]. An estimated 4.29 million new cancer cases and 2.81 million cancer deaths occurred in China in 2015 [12]. There have been more than 30 hospice centers sponsored by Li Ka Shing Foundation in mainland China, which provide home-based palliative care services for around 16 thousand economically disadvantaged patients with advanced cancer living in urban and remote rural villages each year [13]. Prognostic tools are necessary for the home-based palliative care (HPC) setting because palliative care specialists are possible to provide more accurate prognostic information with these tools when communicating with the patient and family.

Several prognostic tools have been developed and validated, which are frequently applied to predict the survival of advanced cancer patients in the hospital palliative care setting [14–22]. However, a blood test is not always available for patients with advanced cancer, especially those in the HPC setting [23]. Comparing with other validated prognostic tools, the Palliative Prognostic Index (PPI) consisting of five independently predictive variables may be more suitable for advanced cancer patients in the HPC setting, for which does not require any invasive procedure [23–25]. Previous studies reported that the PPI had a low sensitivity for patients with advanced cancer in the HPC setting [25–30]. However, there is no study to support the validity of the PPI in the HPC setting in mainland China [24, 25]. Recently, Takeshi et al. [31] reported the development of the Performance Status–Based Palliative Prognostic Index (PS-PPI), a brief version of the PPI, in which the performance status was based on the Eastern Cooperative Oncology Group (ECOG) PS instead of the Palliative Performance Scale (PPS). The sensitivity of the PS-PPI for advanced cancer patients was higher than that of the PPI. Further validations for the PS-PPI in other palliative care settings are needed. Besides, the Chinese Prognosis Scale (ChPS), the first prognostic scale in mainland China, was developed based on patients in the HPC setting sponsored by Li Ka Shing Foundation in Shanghai, China by Zhou et al. [10]. However, the ChPS has not been fully validated by other research teams.

It is still uncertain whether the three prognostic tools are suitable for patients with advanced cancer in the HPC setting in mainland China. Therefore, the purpose of the present study was to validate and compare the predictive value of the PPI, PS-PPI and ChPS for patients with advanced cancer in the HPC setting in mainland China.

## Methods

This retrospective observational study was conducted on patients consecutively admitted to the hospice center of Yuebei People’s Hospital sponsored by the Li Ka Shing Foundation between January 2014 and December 2018. In this study, patients who satisfied the following criteria were included: (1) at least 18 years old; (2) must be diagnosed with locally extensive or metastatic advanced cancer in a “high-level hospital”, including hematological neoplasm; (3) agree to participate in palliative home care service. Patients who terminated the services halfway or data missing were excluded. Patients’ demographic information (age, gender, site of primary cancer and metastatic disease and survival time) and clinical characteristics (performance status, symptoms, and signs) were assessed and recorded by a palliative care team, consisting of 2 physicians, 2 specialist nurses, and 1 social worker at the first consultation. All patients were followed up by home visits or phone calls on a regular basis until the end of the service when patients passed away. The calculation of the survival time was from the date of the first assessment until the date of death. The study was approved by the ethical review board of Yuebei People’s Hospital Affiliated to Shantou University Medical College (KY-2019-024) and was performed according to the ethical standards laid down in the 1964 Declaration of Helsinki and its later amendments or comparable ethical standards. Written informed consent was obtained from all individual or guardian participants.

### Chinese prognosis scale

The ChPS was initially developed by Zhou et al. in 2009 to predict the survival of advanced cancer patients in the HPC setting [10]. The ChPS score was calculated by summing the scores of ten prognostic factors: weight loss, nausea, dysphagia, dyspnea, edema, cachexia, dehydration, gender, KPS (Karnofsky Performance Status) scores, and QOL (Quality of Life) scores [32]. [33] The range of ChPS scores is from 0 (no altered variables) to 124 (maximal altered variables). Patients were classified into 2 risk groups according to the original article: group A included patients with ChPS scores ≤28 and group B with ChPS scores > 28. A ChPS score of more than 28 predicts survival of less than 90 days, and a score of less than or equal to 28 predicts survival of 90 to 180 days.

### Palliative prognostic index

The PPI was initially developed by Morita et al. in 1999 to predict the survival of advanced cancer patients in palliative care units [24]. The PPI score was calculated by summing the scores of five independently predictive variables: PPS scores, oral intake, edema, dyspnea at rest, and delirium [21]. The PPS scores in the present study were transferred from the KPS scores. KPS scores of 10–100 corresponded to PPS scores of 10–100, respectively [34]. The range of PPI scores is from 0 to 15. Patients were classified into 3 risk groups according to the original article: group A (0.0–4.0), group B (4.1–6.0), and group C (6.1–15.0). Patients with a PPI score of more than 6 survive less than 21 days, and with a score of more than 4 survive less than 42 days.

### Performance status–based palliative prognostic index

Using the Eastern Cooperative Oncology Group (ECOG) PS to assess advanced cancer patients’ performance status instead of the Palliative Performance Scale (PPS), the PS-PPI was recently developed by Takeshi et al. in 2016 to predict the survival of advanced cancer patients [31]. The PS-PPI score was calculated by summing the scores of the ECOG PS scores, oral intake, delirium, dyspnea at rest, and edema [35]. The ECOG PS scores in the present study were transferred from the KPS scores. KPS scores of 100, 90–80, 70–60, 50–40, and 30–10 corresponded to ECOG PS scores of 0, 1, 2, 3, and 4, respectively [34]. The range of PS-PPI scores is from 0 to 15. Patients were classified into 3 risk groups according to the original article: group A (0.0–4.0), group B (4.1–6.0), and group C (6.1–15.0). A PS-PPI score of more than 6 predicts survival of less than 21 days, and a score of more than 4 predicts survival of less than 42 days.

### Statistical analysis

General characteristics of patients and variables of the three prognostic tools were summarized and analyzed. Survival curves were estimated using the Kaplan-Meier method, and the log-rank test was used to compare survival times among each risk group of these three prognostic tools. The area under the receiver operating characteristic curve (AUROC) was calculated to determine the accuracy of the three prognostic tools for predicting survival time within 7, 14, 21, 30, 42, 60, 90, 120, 150, and 180 days. The AUROC of the PPI, PS-PPI and ChPS were compared respectively based on the DeLong method [36]. Sensitivity, specificity, positive predictive value (PPV), negative predictive value (NPV), and overall accuracy (OA) were calculated for prediction of 21-day, 42-day, and 90-day survival using the best cutoff score, which was decided by Youden index. In all analyses, *P* < 0.05 was defined as significance. Survival time was presented as median (95% CI, confidence intervals), and continuous data was presented as mean (SD, standard deviation). MedCalc version 18.2.1 (MedCalc Software, Ostend, Belgium) was used to compare the AUROC among the three prognostic tools. Additional statistical analyses were carried out with IBM SPSS Statistics Version 22.0.0.0 (SPSS Inc., Chicago, IL) and GraphPad Prism Version 8.0.2 (GraphPad Software, San Diego, CA).

## Results

### General characteristics of patients

A total of 1863 patients were included in the study. Table 1 shows background information of patients in detail. The mean age of patients was 61.5 ± 12.64 years, and males accounted for 62.6% of the sample. The most prevalent primary cancer sites of the patients were as follows: lung (31.6%), liver (14.9%), and colon/rectum/small intestine (12.1%). The median survival time of patients was 52 days.

**Table 1:** General Characteristics of Patients (*N* = 1863)

Characteristics	Mean ± SD or *N* (%)
Age, years	61.5 ± 12.64
Gender	
Male	1166 (62.6)
Female	697 (37.4)
Site of primary cancer	
Lung	590 (31.7)
Esophagus/stomach	171 (9.2)
Colon/rectum/small intestine	226 (12.1)
Liver	277 (14.9)
Pancreas	60 (3.2)
Biliary system	36 (1.9)
Breast	84 (4.5)
Kidney/renal pelvis/ureter/bladder/prostate	56 (3)
Ovary/uterus	103 (5.5)
Head and neck (incl thyroid)	141 (7.6)
Blood (leukaemia/myeloma/lymphoma)	27 (1.4)
Central nervous system	10 (0.5)
Soft tissue (Sarcoma)	15 (0.8)
Unknown	29 (1.6)
Other	45 (2.4)
Metastatic site	
Any site	1590 (85.3)
Liver	438 (23.5)
Lung	382 (20.5)
Bone	512 (27.5)
Central nervous system	133 (7.1)
Survival time (day)^a^	52 (49.0–56.0)
<21	385 (20.7)
<42	773 (41.5)
<90	1225 (65.8)
<180	1552 (83.3)

*Abbreviation*: *SD* standard deviation

^a^ Data were expressed as median (95% confidence intervals)

### Survival analysis of the three prognostic tools

**Fig. 1:**
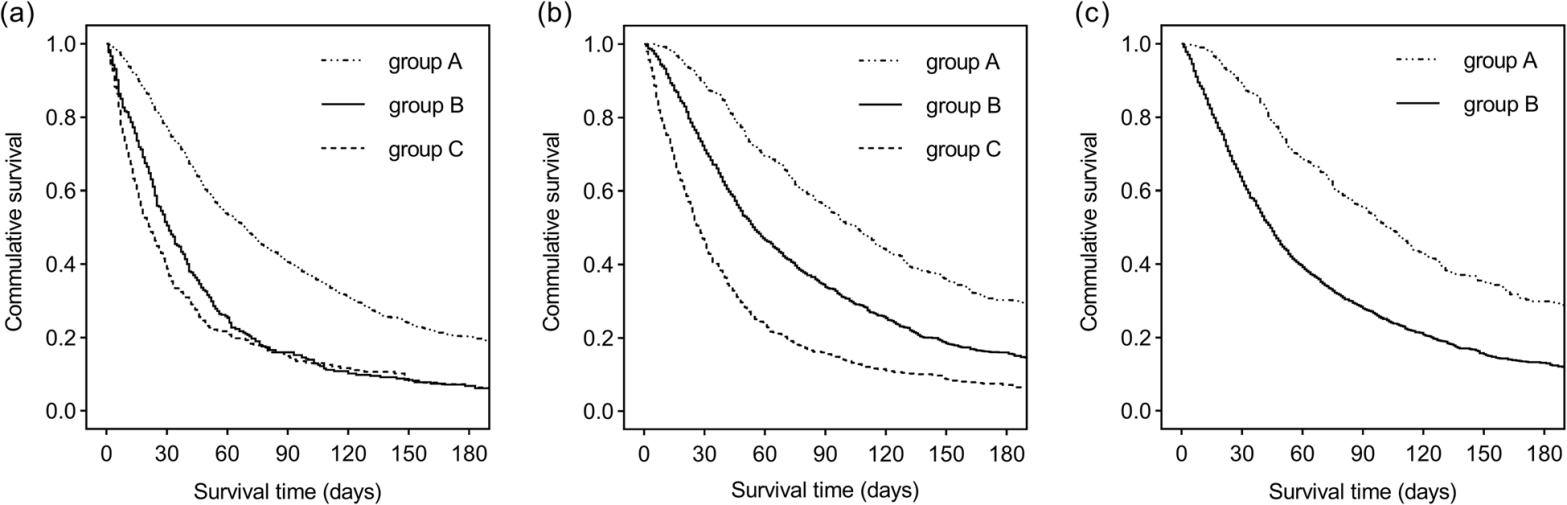
Kaplan-Meier survival curves of the risk groups categorized by the three prognostic tools. **a**, Palliative Prognostic Index: group A (0.0–4.0), group B (4.1–6.0), and group C (6.1–15.0). **b**, Performance Status-Based Palliative Prognostic Index: group A (0.0–4.0), group B (4.1–6.0), and group C (6.1–15.0). **c**, Chinese Prognostic Scale: group A (0.0–28.0) and group B (28.1–124.0). Log-rank tests were all significant for the 3 prognostic tools (*P*<0.001), except survival time between group B and group C (*P*=0.089) in Palliative Prognostic Index.

Particular variables of the three prognostic tools are shown in Table 2. Risk groups of the three prognostic tools and median survival time of patients are shown in Table 3. The values for median survival and relative 95% CI and distribution for the three risk groups of the PPI were 69 days (95%CI 63–74 days) in group A (0.0–4.0; 73.1%), 31 days (95%CI 26–37 days) in group B (4.1–6.0; 15.8%), 22 days (95%CI 17–27 days) in group C (6.1–15.0; 11.1%); the values for median survival and relative 95% CI and distribution for the three risk groups of the PS-PPI were 105 days (95%CI 93–116 days) in group A (0.0–4.0; 21.8%), 55 days (95%CI 50–60 days) in group B (4.1–6.0; 51.0%), 27 days (95%CI 24–30 days) in group C (6.1–15.0; 27.2%); the values for median survival and relative 95% CI and distribution for the two risk groups of the ChPS were 103 days (95%CI 93–114 days) in group A (0.0–28.0; 21.5%), 44 days (95%CI 41–47 days) in group B (28.1–124.0; 78.5%). The survival times of the risk groups of the PS-PPI and ChPS were significantly different from each other (*P* < 0.001). Nevertheless, the survival times of the risk groups of the PPI were not significantly different from each other (*P* = 0.089). Kaplan- Meier survival curves are shown in Fig. 1.

**Table 2:** Variables of the Three Prognostic Tools

PPI and PS-PPI		ChPS	
Variables	*N* (%)	Variables	*N* (%)
PPS		Weight loss	
10–20	2 (0.1)	No	22 (1.2)
30–50	1382 (74.2)	Yes	1841 (98.8)
≥60	479 (25.7)	Nausea	
Oral intake		No	1347 (72.3)
Normal	15 (0.8)	Yes	516 (27.7)
Moderately reduced	1679 (90.1)	Dysphagia	
Severely reduced	169 (9.1)	No	1775 (95.3)
Edema		Yes	88 (4.7)
No	1582 (84.9)	Dyspnea	
Yes	281 (15.1)	No	1683 (90.3)
Dyspnea at rest		Yes	180 (9.7)
No	1683 (90.3)	Edema	
Yes	180 (9.7)	No	1582 (84.9)
Delirium		Yes	281 (15.1)
No	1825 (98.0)	Gender	
Yes	38 (2.0)	Male	1166 (62.6)
ECOG PS		Female	697 (37.4)
0–1	2 (0.1)	Cachexia	
2	477 (25.6)	No	1745 (93.7)
3–4	1384 (74.3)	Yes	118 (6.3)
		Dehydration	
		No	1772 (95.1)
		Yes	91 (4.9)
		QOL	
		> 40	37 (2.0)
		31–40	1224 (65.7)
		0–30	602 (32.3)
		KPS	
		> 70	2 (0.1)
		70	30 (1.6)
		60	447 (24.0)
		≤50	1384 (74.3)

*Abbreviations*: *PPI* Palliative Prognostic Index, *PS-PPI* Performance Status-Based Palliative Prognostic Index, *ChPS* Chinese Prognostic Scale, *PPS* Palliative Performance Scale, *ECOG PS* Eastern Cooperative Oncology Group Performance Status, *KPS* Karnofsky Performance Status, *QOL* Quality of Life

**Table 3:** Median Survival Time of the Three Prognostic Tools

PPI			PS-PPI			ChPS		
Risk groups (total scores)	Number of patients (%)	Median survival (days)(95%CI)	Risk groups (total scores)	Number of patients (%)	Median survival (days)(95%CI)	Risk groups (total scores)	Number of patients (%)	Median survival (days)(95%CI)
A (0.0–4.0)	1361 (73.1)	69 (63–74)	A (0.0–4.0)	407 (21.8)	105 (93–116)	A (0.0–28.0)	401 (21.5)	103 (93–114)
B (4.1–6.0)	295 (15.8)	31 (26–37)	B (4.1–6.0)	950 (51.0)	55 (50–60)	B (28.1–124.0)	1462 (78.5)	44 (41–47)
C (6.1–15.0)	207 (11.1)	22 (17–27)	C (6.1–15.0)	506 (27.2)	27 (24–30)			

*Abbreviations CI*, confidence intervals; *PPI* Palliative Prognostic Index; *PS-PPI* Performance Status-Based Palliative Prognostic Index; *ChPS* Chinese Prognostic Scale

### Accuracy of the three prognostic tools

**Fig. 2:**
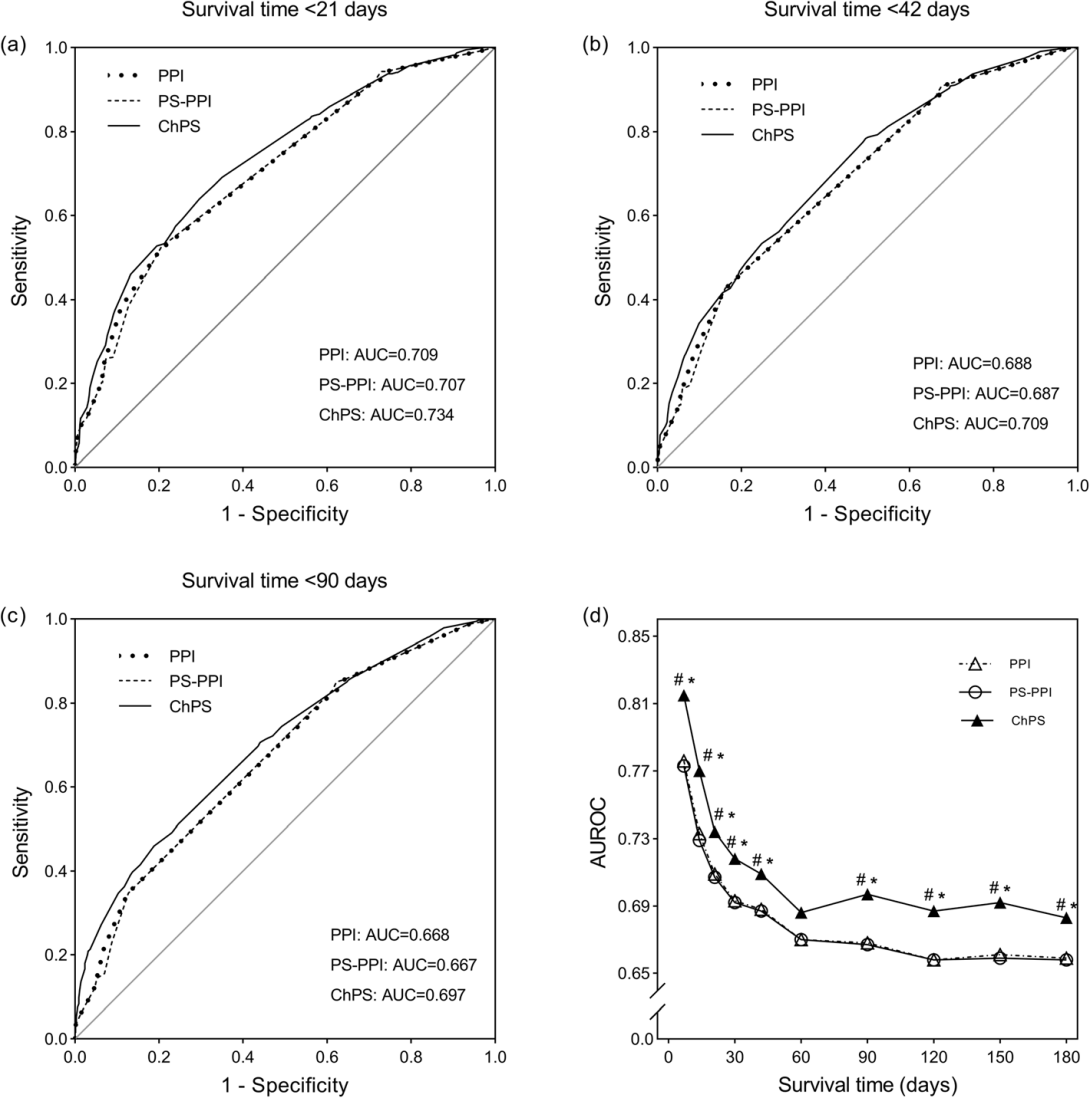
Comparison of the AUROC for 21-day, 42-day, and 90-day survival among the three prognostic tools. **a**, The AUROC values for 21-day survival were 0.709 for the PPI, 0.707 for the PS-PPI, and 0.734 for the ChPS. The best cutoff scores for 21-day survival were 4.5 for the PPI, 6 for the PS-PPI, and 39 for the ChPS. **b**, The AUROC values for 42-day survival were 0.688 for the PPI, 0.687 for the PS-PPI, and 0.709 for the ChPS. The best cutoff scores for 42-day survival were 4 for the PPI, 6 for the PS-PPI, and 35 for the ChPS. **c**, The AUROC values for 90-day survival were 0.668 for the PPI, 0.667 for the PS-PPI, and 0.697 for the ChPS. The best cutoff scores for 90-day survival were 4 for the PPI, 4.5 for the PS-PPI, and 38 for the ChPS. **d**, Comparison of the AUROC values for 7-, 14-, 21-, 30-, 42-, 60-, 90-, 120-, 150- and 180-day survival among the three prognostic tools. # *AUROC are significantly higher in the ChPS than both PPI and PS-PPI. * *P* < 0.05, ChPS versus PPI; # *P* < 0.05, ChPS versus PS-PPI. Abbreviation: AUROC, Area Under the Receiver Operating Characteristic Curve; PPI, Palliative Prognostic Index; PS-PPI, Performance Status-Based Palliative Prognostic Index; ChPS, Chinese Prognostic Scale All tables could be placed at the end of Results during production.

Receiver operating characteristic curves for 21-, 42-, 90- and 180-day survival of the three prognostic tools were compared (Fig. 2 a, b, c). The AUROC values for 7-, 14-, 21-, 42-, 60-, 90-, 120-, 150- and 180-day survival were 0.776, 0.733, 0.709, 0.693, 0.688, 0.67, 0.668, 0.658, 0.661 and 0.659, respectively for the PPI; were 0.773, 0.729, 0.707, 0.692, 0.687, 0.67, 0.667, 0.658, 0.659 and 0.658, respectively for the PS-PPI; were 0.815, 0.77, 0.734, 0.718, 0.709, 0.686, 0.697, 0.687, 0.692 and 0.683, respectively for the ChPS. The AUROC of the ChPS was significantly higher than that of the PPI and PS-PPI for 7-, 14, 21-, 42-, 90-, 120-, 150- and 180-day survival (*P* < 0.05). The AUROCs of the PPI and PS-PPI were not significantly different from each other (*P* > 0.05) (Fig. 2 d). The best cutoff scores for 21-day survival were 4.5 for the PPI, 6 for the PS-PPI, and 39 for the ChPS. The best cutoff scores for 42-day survival were 4 for the PPI, 6 for the PS-PPI, and 35 for the ChPS. The best cutoff scores for 90-day survival were 4 for the PPI, 4.5 for the PS-PPI, and 38 for the ChPS. Besides, the sensitivity, specificity, PPV, NPV values and OA of the three prognostic tools are showed in Table 4.

**Table 4:** Accuracy of the Three Prognostic Tools

	Cutoff	Sensitivity (%, 95CI)	Specificity (%, 95CI)	PPV (%, 95CI)	NPV (%, 95CI)	OA (%, 95CI)
21 days						
PPI	4.5 ^a^	52.0 (46.8–57.0)	79.6 (77.5–81.7)	39.9 (36.6–43.3)	86.4 (85.1–87.6)	73.9 (70.1–77.9)
	5	38.2 (33.3–43.2)	89.0 (87.3–90.6)	47.6 (42.8–52.4)	84.7 (83.6–85.7)	78.5 (74.5–82.6)
	6	26.2 (21.9–30.9)	92.8 (91.4–94.1)	48.8 (42.6–55.0)	82.9 (82.0–83.7)	79.0 (75.0–83.2)
PS-PPI	6 ^a^	52.5 (47.3–57.6)	79.4 (77.3–81.5)	40.0 (36.7–43.3)	86.5 (85.2–87.7)	73.8 (70.0–77.9)
	6.5	38.4 (33.6–43.5)	87.3 (85.5–89.9)	44.0 (39.6–48.6)	84.5 (83.4–85.5)	77.2 (73.3–81.3)
	7	26.2 (21.9–30.9)	90.9 (89.4–92.3)	43.0 (37.4–48.7)	82.6 (81.6–83.4)	77.5 (73.6–81.6)
ChPS	38	67.3 (62.3–71.9)	66.9 (64.4–69.2)	34.6 (32.3–36.9)	88.7 (87.1–90.1)	67.0 (63.3–70.8)
	39 ^a^	63.9 (58.9–68.7)	70.4 (68.0–72.8)	36.0 (33.6–38.6)	88.2 (86.7–89.6)	69.1 (65.4–73.0)
	40	59.2 (54.1–64.2)	74.4 (72.1–76.6)	37.6 (34.8–40.4)	87.5 (86.1–88.8)	71.3 (67.5–75.2)
42 days						
PPI	3.5	88.1 (85.6–90.3)	33.9 (31.1–36.8)	48.6 (47.4–49.9)	80.1 (76.5–83.2)	56.4 (53.1–59.9)
	4 ^a^	42.4 (38.9–46.0)	84.0 (81.7–86.2)	65.3 (61.7–68.8)	67.3 (65.8–68.7)	66.7 (63.1–70.5)
	4.5	42.3 (38.8–45.9)	84.0 (81.7–86.2)	65.3 (61.6–68.8)	67.3 (65.8–68.7)	66.7 (63.1–70.5)
PS-PPI	4	91.2 (89.0–93.1)	31.1 (28.4–34.0)	48.5 (45.9–51.1)	83.3 (79.2–86.7)	56.0 (52.7–59.5)
	5	88.6 (86.2–90.8)	33.7 (30.9–36.6)	48.7 (47.4–49.9)	80.7 (77.1–83.8)	56.5 (53.1–60.0)
	6 ^a^	42.7 (39.2–46.3)	83.9 (81.5–86.0)	65.2 (61.6–68.7)	67.4 (65.9–68.8)	66.8 (63.2–70.7)
ChPS	34	78.8 (75.7–81.6)	49.9 (46.9–52.9)	52.7 (51.0–54.5)	76.8 (74.1–79.4)	61.9 (58.4–65.6)
	35 ^a^	78.5 (75.5–81.4)	50.5 (47.4–53.5)	52.9 (51.1–54.6)	76.7 (74.0–79.2)	62.1 (58.6–65.8)
	36	77.6 (74.5–80.5)	51.1 (48.1–54.1)	53.0 (51.2–54.7)	76.3 (73.6–78.8)	62.1 (58.6–65.8)
90 days						
PPI	3.5	82.5 (80.2–84.5)	38.7 (34.9–42.6)	72.1 (70.7–73.4)	53.5 (49.6–57.3)	67.5 (63.9–71.4)
	4 ^a^	34.5 (31.9–37.3)	87.6 (84.8–90.1)	84.3 (81.1–87.0)	41.1 (39.9–42.3)	52.7 (49.5–56.1)
	4.5	34.5 (31.8–37.2)	87.6 (84.8–90.1)	84.2 (81.1–86.9)	41.0 (39.8–42.3)	52.7 (49.5–56.1)
PS-PPI	4	85.6 (83.5–87.5)	36.1 (32.3–39.9)	72.0 (70.7–73.2)	56.5 (52.3–60.7)	68.6 (64.9–72.5)
	4.5 ^a^	85.1 (82.9–87.0)	37.8 (34.0–41.7)	72.4 (71.1–73.7)	56.8 (52.7–60.9)	68.9 (65.2–72.8)
	5	83.0 (80.8–85.1)	38.7 (34.9–42.6)	72.2 (70.9–73.5)	54.3 (50.4–58.2)	67.8 (64.2–71.7)
ChPS	28	86.1 (84.1–88.0)	34.0 (30.3–37.8)	71.5 (70.2–72.7)	56.1 (51.7–60.4)	68.3 (64.6–72.1)
	33	71.4 (68.8–73.9)	54.6 (50.6–58.5)	75.1 (73.3–76.8)	49.9 (47.0–52.7)	65.6 (62.0–69.4)
	38 ^a^	49.1 (46.3–52.0)	77.0 (73.5–80.2)	80.4 (77.9–82.7)	44.1 (42.4–45.8)	58.7 (55.2–62.3)

*Abbreviations*: *CI* confidence intervals, *PPV* Positive Predictive Value, *NPV* Negative Predictive Value, *OA* Overall Accuracy, *PPI* Palliative Prognostic Index, *PS-PPI* Performance Status-Based Palliative Prognostic Index; *ChPS* Chinese Prognostic Scale. ^a^ Data were expressed as the best cutoff score based on Youden index (Youden index = sensitivity+ specificity- 1)

## Discussion

In the present study, the three prognostic tools were validated and compared simultaneously for predicting the survival of patients with advanced cancer in mainland China, especially for patients who received home-based palliative care services. Our results indicate that the ChPS is more suitable for advanced cancer patients in the HPC setting than the PPI and PS-PPI.

The ChPS was initially developed for predicting the survival of advanced cancer patients in the HPC setting in China [10]. Group B (total scores 28.1–124.0) in ChPS exhibited a significantly shorter survival time than group A (total scores 0.0–28.0) in the present study, which is consistent with the results of Zhou et al. [10] The overall accuracy for predicting the survival of less than 90 days with a ChPS score of 28 was 68.3%, which is similar to the outcome from the original article that the overall accuracy of the testing set was 65.4% [10]. In the present study, the AUROC of the ChPS was significantly higher than that of the PPI and PS-PPI for 7-, 14, 21-, 42-, 90-, 120-, 150- and 180-day survival, and the sensitivity and PPV of the ChPS for predicting 90-day survival were 86.1 and 71.5%, respectively. However, the ChPS had low specificity and NPV in the present study. Low NPV indicated that there were some patients with ChPS scores (0.0–28.0) who lived less than 90 days. In practice, false-positive predictions are more critical than false-negative predictions because the predicted survival time being longer than the actual survival time may induce the problem in communication between clinicians and patients or their family for decision making [37, 38]. Based on this view, the ChPS could be utilized as a screening tool for prognostication because of its high sensitivity and PPV, which is a prerequisite for a useful screening tool. Besides, the classification of primary cancer was included in some scoring systems in previous studies [39–41]. Patients with different types of cancer may have individual survival time because primary cancer is considered a significant factor related to survival time. In addition, previous studies reported that the specific site of metastasis was associated with survival time [42–44]. Yin et al. [42] reported that liver metastasis was regarded as an independent predictor related to poor prognosis for patients with cervical cancer. Another study reported that metastatic renal cell carcinoma patients with isolated liver metastases seemed to have worse outcomes [43]. However, the primary site of the tumor and the specific site of metastasis were not considered in the ChPS. Further studies are needed to confirm that the accuracy of the ChPS could be improved by including factors relative to the primary site of the tumor and the particular site of metastasis.

In a retrospective study, Hamano et al. [27] suggested that the PPI might not be suitable as a screening tool for poor prognosis patients with relatively good performance status in the home care setting considering its low sensitivity, but might be suitable for predicting survival longer than 21 days because of its high specificity. A further prospective study supported this finding [29]. A similar outcome was presented in our study that the sensitivity for 21-day and 42-day survival of the PPI was lower than that of the original article when the cutoff score was set at 6.0 and 4.0, respectively, and the PPI had a high specificity with the same cutoff scores. The discrepancy of the sensitivity may be attributed to differences between the patients. The low sensitivity in the HPC setting could be interpreted by the lowest prevalence for PPI > 6 (11.1%), which indicated that patients with advanced cancer in the HPC setting may be in a better general condition and have fewer complications [10, 45]. Characteristics related to the survival of patients were different for various studies [29, 46]. The median survival time of the patients in the present study was 52 days, whereas Morita et al. [24] reported 27 days, and Maltoni et al. [30] reported 22 days in the hospice setting. In addition, the prevalence of severely reduced oral intake, edema, dyspnea at rest, and delirium was lower than those in the original article. Some scholars hold the view that a one-shot PPI assessment might not be accurate enough as a prognostic tool because patients’ clinical features changed dynamically during the end-of-life trajectory [47–49]. Arai et al. [49] reported a retrospective cohort study that reassessment of the PPI was necessary because of the change in the PPI as an important and independent factor associated with the survival of advanced cancer patients. Another previous study reported by Kao et al. [48] showed that the combination of initial PPI and score change was more accurate to predict the actual prognosis. Further studies are needed to modify the PPI for advanced cancer patients in the HPC setting.

In the previous study, [31] the PS-PPI was as accurate as the PPI to predict the survival of advanced cancer patients, which paralleled our findings that the AUROCs of the PPI and PS-PPI were not significantly different from each other. Survival time among the three risk groups of the PS-PPI was significantly different from each other. However, survival time between group B (4.1–6.0) and group C (6.1–15.0) in PPI was not significantly different from each other, which is not in accordance with previous findings [30, 50]. One possible reason is that patients with PPS scores (30–50) accounted for 74.2% in the present study, which indicated that the performance status of patients might be overestimated by the physicians [10]. Another reason is that patients with moderate performance status could not be distinguished precisely by the KPS. The difference between the PPI and PS-PPI is that ECOG PS is used to take the place of PPS for performance status assessment. Performance status has been found to be strongly correlated with survival time in previous studies [10, 51–54]. The European Association for Palliative Care has recommended the performance status as significant prognostic factors [1]. Myers et al. [55] reported that the ECOG scale, PPS, and KPS have a highly significant linear correlation. Another study reported by Chow et al. [56] suggested that there was a notable correlation of performance status scores among the ECOG scale, PPS, and KPS, and with no one tool statistically superior to others. In the present study, the KPS-to-ECOG and KPS-to-PPS conversion were based on the formula reported by Ma et al. [34] Thus, further studies are needed to compare the PPI and PS-PPI for advanced cancer patients in the HPC setting.

Some other prognostic tools without blood test have been validated with good feasibility and accuracy in the HPC setting, such as the PiPS-A. The PiPS-A composed of thirteen factors was considered to be very useful and effective when laboratory results are unavailable [23]. Besides, Kim et al. also drew a similar conclusion [57]. However, the Karnofsky Performance Status (KPS) scores and the Quality of Life (QOL) scores are mainly used to assess the performance status of patients with advanced cancer in the HPC setting in mainland China, especially in the hospice center sponsored by the Li Ka Shing Foundation. Unfortunately, limited to the retrospective study, the global health status of patient, one of the basic prognostic parameters of the PiPS-A, could not be evaluated through the data provided by the hospice center. Hence, further studies are needed to validate the PiPS-A for patients with advanced cancer in the HPC setting in mainland China.

This study has some limitations. First, our study was carried out retrospectively and included only economically disadvantaged patients from a single institution, which may not be representative of patients with advanced cancer in the HPC setting in mainland China and worldwide. Second, ECOG PS scores and PPS scores were both transferred from KPS scores, which may affect the accuracy of the PPI and PS-PPI. Third, clinical characteristics of patients might be recorded in mistake without standardized specific assessment tools in a retrospective study. Notwithstanding these limitations, a large number of advanced cancer patients in the HPC setting were included in the present study. Meanwhile, the three prognostic tools we selected do not require blood tests and complicated calculations.

## Conclusions

The present study demonstrated that the ChPS is more suitable than the PPI and PS-PPI for patients with advanced cancer in the HPC setting. More researches are needed to verify the predictive value of the ChPS, PPI, and PS-PPI in HPC settings in the future.

## Data Availability

The datasets used and/or analysed during the current study are available from the corresponding author on reasonable request.

## Abbreviations

HPC: Home-Based Palliative Care
PPI: Palliative Prognostic Index
PS-PPI: Performance Status–Based Palliative Prognostic Index
ChPS: Chinese Prognosis Scale
ECOG: Eastern Cooperative Oncology Group
PPS: Palliative Performance Scale
KPS: Karnofsky Performance Status
QOL: Quality of Life
AUROC: The Area Under the Receiver Operating Characteristic Curve
PPV: Positive Predictive Value
NPV: Negative Predictive Value
CI: Confidence Intervals
SD: Standard Deviation
